# Nocturnal Blood Pressure Fluctuations in Patients with Rapid Eye Movement-Related Obstructive Sleep Apnea

**DOI:** 10.3390/jcm10215023

**Published:** 2021-10-28

**Authors:** Hajime Kumagai, Hiroyuki Sawatari, Tetsuro Hoshino, Noriyuki Konishi, Yuka Kiyohara, Kengo Kawaguchi, Hiroko Tsuda, Yoko Haseda, Ryujiro Sasanabe, Toshiaki Shiomi

**Affiliations:** 1 Department of Sleep Medicine, Graduate School of Biomedical and Health Sciences, Hiroshima University, Hiroshima 7348553, Japan; hoshino.tetsurou.299@mail.aichi-med-u.ac.jp (T.H.); h8k2nk@gmail.com (N.K.); ykiyo@hiroshima-u.ac.jp (Y.K.); fukutsu@hiroshima-u.ac.jp (K.K.); htsuda@dent.kyusyu-u.ac.jp (H.T.); shomi21@hiroshima-u.ac.jp (T.S.); 2 Hiroshima Minato Clinic, Hiroshima 7340014, Japan; 3 Department of Sleep Medicine and Sleep Disorders Center, Aichi Medical University Hospital, Aichi 4801195, Japan; sawatari@hiroshima-u.ac.jp (H.S.); yoko_h_1104@yahoo.co.jp (Y.H.); sasanabe@aichi-med-u.ac.jp (R.S.); 4 Department of Perioperative and Critical Care Management, Graduate School of Biomedical and Health Sciences, Hiroshima University, Hiroshima 7348553, Japan

**Keywords:** rapid eye movement-related obstructive sleep apnea, pulse transit time, non-dipping pattern, systolic blood pressure variability

## Abstract

Rapid eye movement-related obstructive sleep apnea (REM-related OSA) is a polysomnographic phenotype. Nocturnal blood pressure (BP) fluctuations remain unclear in patients with REM-related OSA. We studied 27 patients with REM-related OSA, categorized as having REM-apnea-hypopnea index (REM-AHI) ≥ 5/h, REM-AHI/non-REM-AHI ≥ 2, and non-REM-AHI < 15/h. Beat-to-beat systolic BP (SBP) variability and nocturnal SBP fluctuation patterns using pulse transit time (PTT) were investigated. The maximum increase and average nocturnal SBP were significantly higher in males than in females (*p* = 0.003 and *p* = 0.008, respectively). The rate of non-dipping patterns in nocturnal SBP fluctuations was 63% in all patients (males, 70%; females, 50%). Epworth Sleepiness Scale (ESS) and Self-rating Depression Scale (SDS) scores in females were higher than those in males (8.4 ± 6.1 vs. 13.4 ± 5.4 points, *p* = 0.04; 43.8 ± 7.9 vs. 52 ± 11.6 points, *p* = 0.04, respectively). A high proportion of patients with REM-related OSA had a non-dipping pattern. Using PPT, we observed that in patients with REM-related OSA, SBP variability was greater in males. Despite clinical symptoms being slightly more severe in females, nocturnal SBP fluctuations should be considered in male patients with REM-related OSA.

## 1. Introduction

Rapid eye movement-related obstructive sleep apnea (REM-related OSA) is a polysomnographic phenotype in OSA that predominantly or exclusively occurs during rapid eye movement (REM) sleep. REM-related OSA has been observed in approximately 10% to 37% of patients with OSA, and REM-related OSA is common in females and young individuals [1,2,3,4,5]. Patients with OSA have more frequent respiratory events, and their cardiovascular system is likely to be unstable due to respiratory events during REM sleep [6]. Obstructive apnea and hypopnea during REM sleep cause more severe hypoxemia, longer apnea duration, and higher activated sympathetic nerve activity than respiratory events during non-rapid eye movement (NREM) sleep [7,8]. It has also been reported that, after only two weeks, apnea during REM sleep resulted in an increase in blood pressure (BP) and sympathetic nerve activity in rats [9]. Even in healthy individuals, sympathetic nerve activity significantly increases during REM sleep [6]. In patients with OSA, sympathetic nerve activity is high during both awake and sleep periods [6,10].

The relationship between OSA severity and nocturnal systolic BP (SBP) fluctuation patterns has been observed in patients with OSA [11,12]. An association has been reported between REM-related OSA and hypertension, even in males with apnea hypopnea index (AHI) < 10/h [13]. A significant association has been reported between the exacerbation of REM-AHI severity and the prevalence of hypertension, diabetes, and metabolic syndrome [14,15,16]. Since respiratory events frequently occur during REM sleep in patients with REM-related OSA, nocturnal SBP may vary more in these patients than in patients without REM-related OSA due to activated sympathetic nerve activity during REM sleep. Furthermore, REM-related OSA has been reported to be associated with insomnia and depression [17,18,19]. Insomnia and a lower respiratory arousal threshold are more common in female patients with REM-related OSA [18,20].

Due to the frequent occurrence of respiratory events during REM sleep in REM-related OSA, sympathetic nerve activity during REM sleep is likely to increase and nocturnal SBP may vary more than that in patients without REM-related OSA. However, no studies have reported undertaking sex-specific examinations concerning nocturnal SBP fluctuations and beat-to-beat BP variabilities using pulse transit time (PTT) in patients with REM-related OSA.

Therefore, we investigated nocturnal SBP fluctuations and variabilities in patients with REM-related OSA who experienced respiratory events more frequently during REM sleep than during non-REM (NREM) sleep.

## 2. Materials and Methods

### 2.1. Patients

In total, 259 patients diagnosed with OSA (AHI ≥ 5/h) were studied using polysomnography (PSG) at Hiroshima Minato Clinic from March 2018 to April 2021. Patients with REM-related OSA were defined as those who fulfilled all of the following criteria: (i) AHI ≥ 5/h, (ii) REM-AHI/NREM-AHI ≥ 2, and (iii) NREM-AHI < 15/h. Exclusion criteria comprised: (i) patients aged < 20 and >80 years and (ii) patients treated using continuous positive airway pressure (CPAP) or oral appliances. Clinical data were collected from patient medical records. The study protocol was approved by the Institutional Review Board of Aichi Medical University Hospital (approval number 2021-049), and informed consent was obtained from all patients prior to participation.

### 2.2. Questionnaires

All patients completed three questionnaires, namely, the Self-rating Depression Scale (SDS), the Epworth Sleepiness Scale (ESS), and the Pittsburgh Sleep Quality Index (PSQI). The SDS was used to screen for the potential presence of depressive disorders. This scale ranges from 20 to 80 points, and scores > 50 points were considered as indicating depressive disorders [21]. The ESS consists of eight self-administered items and measures a patient’s excessive daytime sleepiness [22]. Higher ESS scores indicate severe daytime sleepiness, and the cutoff value for the ESS in this study was 10/11 points. The PSQI has 19 self-rated individual items and evaluates sleep quality and disorder over a one-month time interval [23], with higher PSQI values indicating poor sleep quality and insomnia.

### 2.3. Polysomnography

All patients underwent overnight PSG using Somnotouch-RESP (Somnomedics, Randersacker, Germany). Electroencephalogram, electrooculogram, and electromyogram data of submental and anterior tibial muscles were recorded, along with electrocardiogram, nasal flow, thoracic and abdominal movement with respiratory effort, oxygen saturation, snoring, finger plethysmogram, and body position data. DOMINO light 1.4.0 (Somnomedics, Randersacker, Germany) was used for recorded data analysis until February 2021, after which DOMINO light 1.5.0. was used from March to April 2021. PSG scoring was performed manually, following the American Academy of Sleep Medicine scoring criteria (version 2.3), by a certified sleep technologist who was blinded to patient information. Apnea was scored when there was a cessation or a drop in flow signal of ≤90% of airflow for at least 10 s. Hypopnea was defined as a ≥30% reduction in airflow for at least 10 s, associated with arousal, or ≥3% oxyhemoglobin desaturation. The AHI was calculated as the average number of apnea and hypopnea events per hour during sleep.

### 2.4. Blood Pressure Measurement

BP was calculated using pulse transit time (PTT) DOMINO software, based on PSG data. BP measurement using PTT is an established indirect BP measurement method. A mutual correlation between oscillometry-based and PTT-based BP measurements has been reported, and the usefulness of the PTT method has also been reported [24,25,26,27]. PTT-BP has certain advantages over 24 h ambulatory BP monitoring, as follows: (i) it enables a noninvasive cuffless measurement and does not disturb sleep, (ii) it enables the calculation of beat-to-beat BP and the detection of dynamic changes in SBP, and (iii) it enables continuous monitoring through recording very short-term BP variability. Therefore, PTT-BP can non-invasively recognize a remarkable increase in BP with OSA during sleep.

PTT was measured at the same time as PSG and calculated as the interval time between the R-wave on electrocardiography and the arrival of the corresponding pulse wave using finger plethysmography. Beat-to-beat SBP and diastolic BP (DBP) values were automatically determined using DOMINO software, based on the initial BP calibration using oscillometry on the upper arm [26,27,28]. Nocturnal BP fluctuation patterns were defined, as follows: (i) dipping ≥ 10% and (ii) non-dipping < 10% fall in SBP from daytime to nighttime sleep. The PTT index was defined as the time per hour of SBP elevation ≥ 12 mmHg in a 30 s interval of sleep.

### 2.5. Statistical Analysis

All statistical analyses were performed using JMP 15.0.0 software (SAS Institute Japan, Tokyo, Japan). Continuous variables are expressed as mean ± standard deviation, and categorical variables are expressed as numbers or percentages. To compare sex differences in terms of demographic, polysomnographic, and PTT parameters, for the continuous variables, *t*-tests and Wilcoxon rank sum tests were conducted for normal distribution and non-normal distribution, respectively. For binary data, a Fisher’s exact test was used to test for differences. All comparisons were two-tailed, and a *p*-value < 0.05 was considered statistically significant. For univariate regression analysis, we estimated the relevant Spearman’s rank correlation coefficient or Pearson correlation coefficient.

## 3. Results

### 3.1. Demographics, Sleep Quality, and PSG Findings

Of 259 patients, 27 patients who had undergone PSG met the definition of REM-related OSA (males, 17 (63.0%); females, 10 (37.0%)), as shown in Table 1. All patients with REM-related OSA slept in a REM sleep for ≥30 min. Of the patients who had undergone PSG, the rates of REM-related OSA in males and females were 8.1% (17/211 patients) and 20.8% (10/48 patients), respectively. The rates of REM-related OSA in females were significantly higher than those in males (*p* = 0.02). Regarding patients with REM-related OSA, the mean age and body mass index (BMI) were 46.0 years and 24.4 kg/m^2^ respectively, and there were no significant differences in age and BMI between males and females (males: 47.9 years, 24.4 kg/m^2^; females: 42.6 years, 24.4 kg/m^2^). Two patients were taking hypnotic medication (male, *n* = 1; female, *n* = 1) and two patients were taking antidepressant medication (male, *n* = 1; female, *n* = 1). Regarding comorbidities, 22.2% of patients with REM-related OSA had hypertension. No significant differences were found in the PTT index, maximum increase in nocturnal SBP, average nocturnal SBP, average nocturnal DBP, and average nocturnal HR, in patients with or without antihypertensive medication.

Of the patients with REM-related OSA, the mean ESS and SDS scores were 10.2 and 46.8 points, respectively (males: ESS = 8.4 points, SDS = 43.8 points; females: ESS = 13.4 points, SDS = 52.0 points, Table 1). The mean ESS and SDS scores in females were higher than the cutoff value and were significantly higher than those in males (*p* = 0.04, both). For males, there were no middle-to-high score (i.e., ≥60 points) groups with SDS, and the average score was within a normal range. The mean PSQI score was 8.3 points (males, 7.9 points; females, 8.8 points). There was no significant difference between males and females, but the mean score in males and females was higher than the cutoff value for PSQI.

Of the patients with REM-related OSA, the mean AHI in males and females was 14.4/h and 11.5/h, respectively (Table 1). NREM-AHI was 10.3/h for males and 8.5/h for females, whereas the REM-AHI was 34.1/h for males and 26.0/h for females. There were no patients with severe sleep disordered breathing (i.e., AHI ≥ 30/h), whereas the REM-AHI was within the severe range, especially for males.

### 3.2. PTT Index and Blood Pressure Using PTT

**Figure 1:**
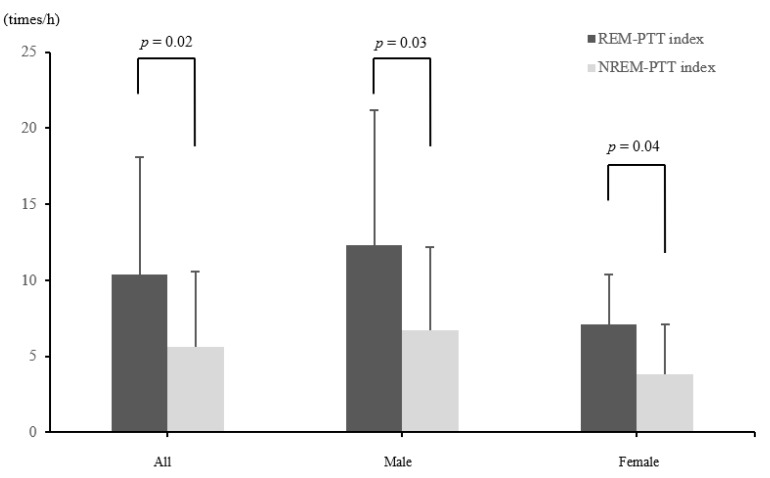
A comparison of PTT indexes during REM and NREM sleep. Continuous variables are expressed as mean ± standard deviation (SD). A comparison of the REM-PTT index and the NREM-PTT index (*p* <0.05) was considered statistically significant. Abbreviations: NREM-PTT index, pulse transit time index during non-rapid eye movement sleep; REM-PTT index, pulse transit time index during rapid eye movement sleep.

The lowest oxygen saturation correlated significantly with the PTT index and maximum SBP increase in all included patients (r = −0.55, *p* = 0.003; r = −0.51, *p* = 0.006, respectively). In male patients, the lowest oxygen saturation also significantly correlated with the PTT index and the maximum SBP increase (r = −0.64, *p* = 0.006; r = −0.59, *p* = 0.0013, respectively). The PTT index and the maximum SBP increase significantly correlated in all included male and female patients (r = 0.75, *p* < 0.0001; r = 0.72, *p* = 0.001; and r = 0.72, *p* = 0.019, respectively). The REM-PTT index was significantly higher than the NREM-PTT index in both male and female groups (*p* = 0.03 and *p* = 0.04 respectively, Figure 1). The PTT index, the REM-PTT index, and the NREM-PTT index did not differ significantly between males and females (Table 2). The average maximum increase in nocturnal SBP was 25.7 mmHg for males and 19.3 mmHg for females (*p* = 0.003). The average nocturnal SBP was 126.9 mmHg for males and 107.1 mmHg for females (*p* = 0.008). The average nocturnal DBP and the average heart rate did not differ significantly between males and females.

### 3.3. Nocturnal Blood Pressure Fluctuations

In patients with REM-related OSA, the most observed nocturnal SBP fluctuation pattern was a non-dipping pattern (*n* = 19, 70.4%), followed by a dipping one (*n* = 8, 29.6%) (Table 2). In particular, a non-dipping pattern was observed in 70% of male patients. Although rising and extreme dipping patterns were not observed in males, rising and extreme dipping patterns were observed in females (20% and 10%, respectively).

## 4. Discussion

This study is the first to report a highly non-dipping pattern in nocturnal SBP fluctuations and greater beat-to-beat SBP variability in patients with REM-related OSA. In addition, females with REM-related OSA might experience more excessive daytime sleepiness and subjective depression than males.

A previous study reported that patients with OSA had a low arousal threshold [29]. REM-related OSA has also been reported to be associated with insomnia and depressive symptoms, especially in females [5,17,18,19,30]. The findings in this study showed that the rate of REM-related OSA in females was significantly higher than that in males, and that females had significantly higher mean ESS and SDS scores than males. This result suggests that female patients with REM-related OSA may have excessive daytime sleepiness and subjective depression. In addition to sex differences, our study findings showed a significant correlation between lowest oxygen saturation and fluctuating BP (i.e., PTT index and maximum SBP increases) in males only. The mechanism of sex differences in terms of elevation of BP due to OSA is unclear; however, previous studies have shown that the prevalence of hypertension increases with the severity of SDB in males, but this trend was not observed in females [31,32]. In a European-based epidemiological study, a significant relationship was found between AHI and the prevalence of hypertension in males only [33], which is consistent with the results of this study.

The PTT index indicates the frequency of beat-to-beat SBP variability within a short time. The PTT index and the maximum SBP increase were significantly correlated. Moreover, the maximum SBP increase was significantly higher in males than in females. Beat-to-beat SBP variability tends to fluctuate largely in severe OSA, with 76% of SBP elevations of >10 mmHg above baseline reported to occur during REM sleep [26]. Although there was no significant difference between males and females in the REM-PTT index and the NREM-PTT index observed in our study, the REM-PTT index was significantly higher than the NREM-PTT index in both males and females. In other words, this suggests that in patients with REM-related OSA, SBP variabilities occur more frequently during REM sleep than during NREM sleep in both males and females. Since increased SBP variability affects various organs, regardless of the BP value [34], patients with REM-related OSA would need to reduce SBP variability.

In this study, the average nocturnal SBP in all patients with REM-related OSA was 119.6 mmHg, which was not high. A previous study reported that as OSA became more severe, the risk of a non-dipping pattern increased in males [11,35,36,37], and that the rate of systolic non-dipping was 33.3% in patients with AHI ≥ 30/h [11,35,36,37]. Another study showed that 48% of patients with OSA had a systolic non-dipping pattern [38]. Furthermore, in a meta-analysis, a non-dipping pattern was found in 59% of patients with OSA, and patients with OSA had a 1.5-fold increased risk of a non-dipping pattern [12]. REM-AHI ≥ 15 has been reported to be significantly associated with non-dipping patterns during REM sleep [35]. In our study, of the patients with REM-related OSA, 70.4% had a non-dipping pattern, which was higher than previously reported for patients with OSA. Furthermore, in males, 94.4% of patients with a non-dipping pattern had REM-AHI ≥ 15/h, and 83.3% of patients had REM-AHI ≥ 30/h. Moreover, the proportion of those with a non-dipping pattern was approximately 2.4-fold higher than that of those with a dipping pattern among the patients with REM-related OSA. These results indicate that a non-dipping pattern occurred in patients with REM-related OSA at a rate equal to or higher than that in patients with OSA who had an already identified non-dipping pattern. AHI in REM-related OSA patients in this study was mild to moderate, less severe than in the severe OSA patients with AHI ≥ 30 previously reported for non-dipping patterns. Nevertheless, the frequency of non-dipping patterns in REM-related OSA patients was higher than that in severe OSA patients. Regarding the frequency of non-dipping patterns, it may be necessary to consider the effects of OSA severity. Moreover, OSA and non-dipping patterns have also been associated with higher risks of hypertension and cardiovascular events [11,12]. Therefore, patients with REM-related OSA and a high non-dipping rate can be expected to have a higher risk of hypertension and cardiovascular events than patients with OSA.

Obstructive apnea and hypopnea during REM sleep result in more severe hypoxemia, longer apnea duration, and greater sympathetic nerve activity than NREM sleep-related respiratory events [7,8]. Mean oxygen desaturation during REM sleep has been reported to be significantly associated with the prevalence of hypertension [12]. Severe OSA with an AHI ≥ 30/h is related to higher SBP and drastic nocturnal BP fluctuations during sleep [26], which may increase the risk of cardiovascular events. Among our patients, the mean AHI was 13.3/h with mild OSA levels, and there was no severe OSA (i.e., AHI ≥ 30/h). However, there were patients with a REM-AHI ≥ 30/h, of whom 85.7% were males. REM-OSA has been significantly and independently associated with the prevalence and incidence of hypertension [12,13]. Compared with patients with a REM-AHI < 5/h, patients with a REM-AHI > 30/h have been reported to have an approximate 2.6 times greater risk of cardiovascular events [39]. Furthermore, the proportion of patients without cardiovascular events, including the presence of hypertension and their survival rate, have been reported to decrease, in line with higher REM-AHI values [39]. Therefore, male patients with a REM-AHI ≥ 30/h are more likely to activate sympathetic nerves and could be considered at high-risk for developing hypertension and cardiovascular events due to a very short-term increase in SBP, a non-dipping pattern of SBP fluctuation, and a lowering of oxygen desaturation. Although the prevalence of REM-related OSA has been reported to decrease with age in females, the prevalence did not change with age in males [4,17]. Thus, the REM-AHI and non-dipping patterns might affect the progression to hypertension and cardiovascular events, especially in male patients with REM-related OSA.

As mentioned above, REM-related OSA might be a high-risk factor for hypertension and cardiovascular events in terms of nocturnal beat-to-beat SBP variabilities, maximum SBP increases, the pattern of SBP fluctuations, and the REM-AHI. Since no correlation was found between beat-to-beat SBP variabilities and patterns of BP fluctuations, this must be considered as another risk factor. Therefore, it is important to evaluate SBP fluctuations as well as high SBP values. In clinical practice, patient follow-up is necessary, with an emphasis on these risk factors.

This study had several limitations. The study sample number was small, and only Japanese patients were included. Due to the small number of patients, multivariate analysis was not performed. When assessing PTT-BP, clinicians need to be aware that postural position, snoring, and arrhythmia during sleep affect PTT-BPs. As a consequence of limiting the study to patients with REM-related OSA, those with severe OSA were not included. Calculating PTT-BP involved only a one-time calibration, although using PTT-BP may enable better assessment of BP variability than an absolute BP value. In addition, we did not compare the results of the questionnaires and nocturnal BP fluctuation data in the patients with non-REM-related OSA because of varied OSA phenotypes (i.e., predominantly central type apnea or predominantly hypopnea). Further careful observation is required to determine whether cardiovascular events are more likely to be induced in patients with REM-related OSA with a non-dipping pattern.

## 5. Conclusions

This is the first study to compare males and females using PTT in patients with REM-related OSA, and we showed that SBP variability was greater in males than in females. A high proportion of non-dipping patterns regarding nocturnal SBP and greater beat-to-beat SBP fluctuations in patients with REM-related OSA was also observed. Therefore, as a feature of REM-related OSA, nocturnal BP fluctuations should be considered in clinical settings.

## Figures and Tables

**Table 1:** Patient demographics, questionnaires, and polysomnography.

	Overall	Males	Females	*p*-Value
Number of patients	27	17	10	-
**Demographics**				
Age (year)	46.0 ± 11.7	47.9 ± 13.1	42.6 ± 8.3	0.2587
BMI (kg/m^2^)	24.4 ± 3.6	24.4 ± 2.6	24.4 ± 5.1	0.7252
Smoking (N)	8	6	2	-
Antihypertensive (N)	6	5	1	-
Hypnotics (N)	2	1	1	-
Antidepressants (N)	2	1	1	-
**Questionnaire**				
ESS (points)	10.2 ± 6.3	8.4 ± 6.1	13.4 ± 5.4	0.04 *
SDS (points)	46.8 ± 10.2	43.8 ± 7.9	52.0 ± 11.6	0.038 *
PSQI (points)	8.3 ± 4.1	7.9 ± 3.9	8.8 ± 4.5	0.6056
**PSG findings**				
Total sleep time (minutes)	395 ± 93	377 ± 111	426 ± 38	0.0926
Sleep efficiency (%)	84.6 ± 8.1	86.0 ± 7.6	82.3 ± 8.8	0.2566
Sleep latency (minutes)	15.0 ± 22.9	8.8 ± 7.5	25.4 ± 35.0	0.0699
REM-sleep latency (minutes)	88.0 ± 47.2	82.3 ± 44.5	97.6 ± 52.4	0.4264
Stage N1 (%)	18.0 ± 6.5	18.7 ± 6.9	16.7 ± 5.8	0.4386
Stage N2 (%)	61.2 ± 7.1	62.0 ± 7.6	59.8 ± 6.2	0.4297
Stage N3 (%)	4.5 ± 5.2	3.0 ± 4.2	7.0 ± 6.0	0.0518
Stage REM (%)	16.3 ± 5.2	16.2 ± 5.1	16.6 ± 5.5	0.8738
AHI (/h)	13.5 ± 4.7	14.7 ± 4.6	11.6 ± 4.4	0.0967
REM-AHI (/h)	31.9 ± 12.1	35.1 ± 12.0	26.4 ± 10.6	0.0687
NREM-AHI (/h)	9.7 ± 3.2	10.5 ± 3.0	8.4 ± 3.1	0.1076
Lowest O_2_ saturation (%)	86.3 ± 4.5	85.6 ± 4.5	87.4 ± 4.5	0.3405
CT90 (minutes)	3.9 ± 10.5	5.7 ± 13.0	0.8 ± 1.5	0.0922
PLMI (/h)	5.1 ± 7.7	5.1 ± 8.7	5.2 ± 6.0	0.9732
Arousal index (/h)	10.9 ± 4.1	10.9 ± 4.0	10.9 ± 4.6	0.9983
REM-arousal index (/h)	10.2 ± 6.2	11.3 ± 7.1	8.4 ± 4.2	0.252
NREM-arousal index (/h)	12.2 ± 7.2	12.8 ± 8.3	11.3 ± 5.2	0.6147

Continuous variables are expressed as mean ± standard deviation (SD). Comparing males and females, a *p*-value of < 0.05 was considered statistically significant. Abbreviations: AHI, apnea hypopnea index; BMI, body mass index; CT90, cumulative time percentage with SpO_2_ < 90%; ESS, Epworth Sleepiness Scale; NREM-AHI, AHI during NREM sleep; NREM-arousal index, arousal index during NREM sleep; PLMI, periodic limb movement index; PSQI, Pittsburgh Sleep Quality Index; REM: rapid eye movement; REM-AHI, AHI during REM sleep; REM-arousal index: arousal index during REM sleep; SDS, Self-rating Depression Scale, * *p* < 0.05.

**Table 2:** Characteristics of the PTT index, PTT-BP, and nocturnal SBP fluctuation patterns.

	Overall	Male	Female	*p*-Value
**PTT index**				
PTT index (/h)	6.5 ± 5.6	7.9 ± 6.4	4.2 ± 3.0	0.183
REM-PTT index (/h)	10.4 ± 7.7	12.3 ± 8.9	7.1 ± 3.3	0.1385
NREM-PTT index (/h)	5.6 ± 5.0	6.7 ± 5.5	3.8 ± 3.3	0.3397
**Blood pressure using PTT**				
Maximum increase in nocturnal SBP (mmHg)	23.3 ± 7.8	25.7 ± 8.9	19.3 ± 2.4	0.0032 *
Average nocturnal SBP (mmHg)	119.6 ± 19.5	126.9 ± 16.4	107.1 ± 18.7	0.0082 *
Average nocturnal DBP (mmHg)	76.3 ± 12.1	79.2 ± 12.4	71.3 ± 10.2	0.0999
Average nocturnal heart rate (beat/min)	67.4 ± 9.3	65.6 ± 7.4	70.6 ± 11.5	0.1798
**Blood pressure fluctuation pattern (N)**				
Dipping	8	5	3	
Non-dipping	19	12	7	

Continuous variables are expressed as mean ± standard deviation (SD). When comparing males and females, a *p*-value < 0.05 was considered statistically significant. Abbreviations: DBP, diastolic blood pressure; NREM, non-rapid eye movement; PTT, pulse transit time; REM, rapid eye movement; REM-PTT index, PTT index during REM sleep; SBP, systolic blood pressure; NREM-PPT, PTT index during NREM sleep, * *p* < 0.05.

## Data Availability

Not applicable.
